# A Text-Book of Anatomy

**Published:** 1900-03

**Authors:** 


A TEXT-BOOK OF ANATOMY.—By American authors. Edited by Frederic H. Gerrish, M. D., Professor of Anatomy in the Medical School of Maine at Bowdoin College. In one magnificent imperial octavo volume of 915 pages with 950 engravings in black and colors. Cloth, $6.50, net; flexible water-proof binding for the dissecting table, $7.00, net; full leather, $7.50, net. Lea Brothers & Co., publishers, Philadelphia and Ne wYork.

America now has a representative treatise on anatomy, and one which may excite justifiable feelings of pride. Stronger reasons than this, however, have led to its creation, for its science is perpetually advancing in discoveries of fact, new views of relationship and improved methods of teaching. Everything of the best this volume strives to embody. Its text is written by professors in leading colleges, is authoritative, uniform, simple and includes all that medical students and practitioners can need, excluding the vastly larger amount of anatomical lore for which there is no known medical application. The illustrations far outnumber and exceed in size and in profusion of colors those in any previous work, and they can well claim to be the most successful series of anatomical pictures in the world. The names of the parts are engraved directly upon them, whereby the nomenclature, position, extent and relations of the various structures are grasped at a glance, and the difficulties inseparable from reference lines and letters avoided. Students especially will be interested in the handsome new flexible water-proof binding, which enables them to lay the book against the cadaver and use it as a dissecting guide, for which it is also adapted by a novel and ingenious arrangement in the text. It can be sponged clean indefinitely without injury. The publishers have endeavored to produce as perfect a book as possible, regardless of its cost to them, and they at once anticipate an extraordinary sale, and assure its realization by joining great value with moderate price.

It will be of special interest to Texas physicians and students to know that a considerable proportion of this volume is the work of our own anatomist, Professor William Keiller, of the Galveston school. T. J. B.

